# Dewatering assessment of sewage sludge: a remaining challenge

**DOI:** 10.1007/s11157-026-09776-4

**Published:** 2026-04-16

**Authors:** Javier Pavez-Jara, Leon Korving, David Jeison, Merle K. de Kreuk

**Affiliations:** 1 Department of Water Management, Delft University of Technology, Stevinweg 1, 2628 CN Delft, Delft, The Netherlands; 2 Escuela de Ingeniería Bioquímica, Pontificia Universidad Católica de Valparaíso, Av. Brasil 2085, 2362803 Valparaíso, Chile; 3 Wetsus, European Centre of Excellence for Sustainable Water Technology, Oostergoweg 9, 8911 MA Leeuwarden, Leeuwarden, The Netherlands

**Keywords:** Dewaterability, Dewatering, Conditioners, Sludge, Filtration, Centrifugation

## Abstract

Improved sewage sludge dewaterability saves costs in biosolids transport and disposal, making reliable dewaterability assessment essential for both research and full-scale process evaluation. However, laboratory-based indexes commonly used to predict dewatering performance often show limited correspondence with full-scale results. Nonetheless, indexes such as capillary suction time (CST), sedimentation and centrifugation methods, specific resistance to filtration (SRF), and mixed dead-end techniques show biases when trying to resemble full-scale results. In our present article, we pose that lack of predictability originates from overlooking all the phenomena involved in the dewatering process. Four critical phenomena are identified to occur during dewaterability: (1) mixing of sludge and conditioner; (2) suspension destabilisation; (3) flocs formation, and (4) compression and expression. By systematically evaluating widely used laboratory indexes this review shows that these methods capture only part of the dewatering process and generally fail to represent the compression and expression stages that ultimately determine the ultimate achievable solids concentration. The analysis highlights sludge compressibility as a critical factor limiting the predictive capacity of conventional indexes. Based on this synthesis, centrifugation, dead-end filtration, and combined centrifugation–filtration approaches are identified as more suitable methods for laboratory-scale assessment because they better represent the compression behaviour of sludge. The review provides a conceptual framework linking dewatering phenomena with experimental assessment methods, supporting the development of improved evaluation strategies and facilitating the testing of emerging, environmentally friendly conditioning technologies.

**Graphical abstract:**

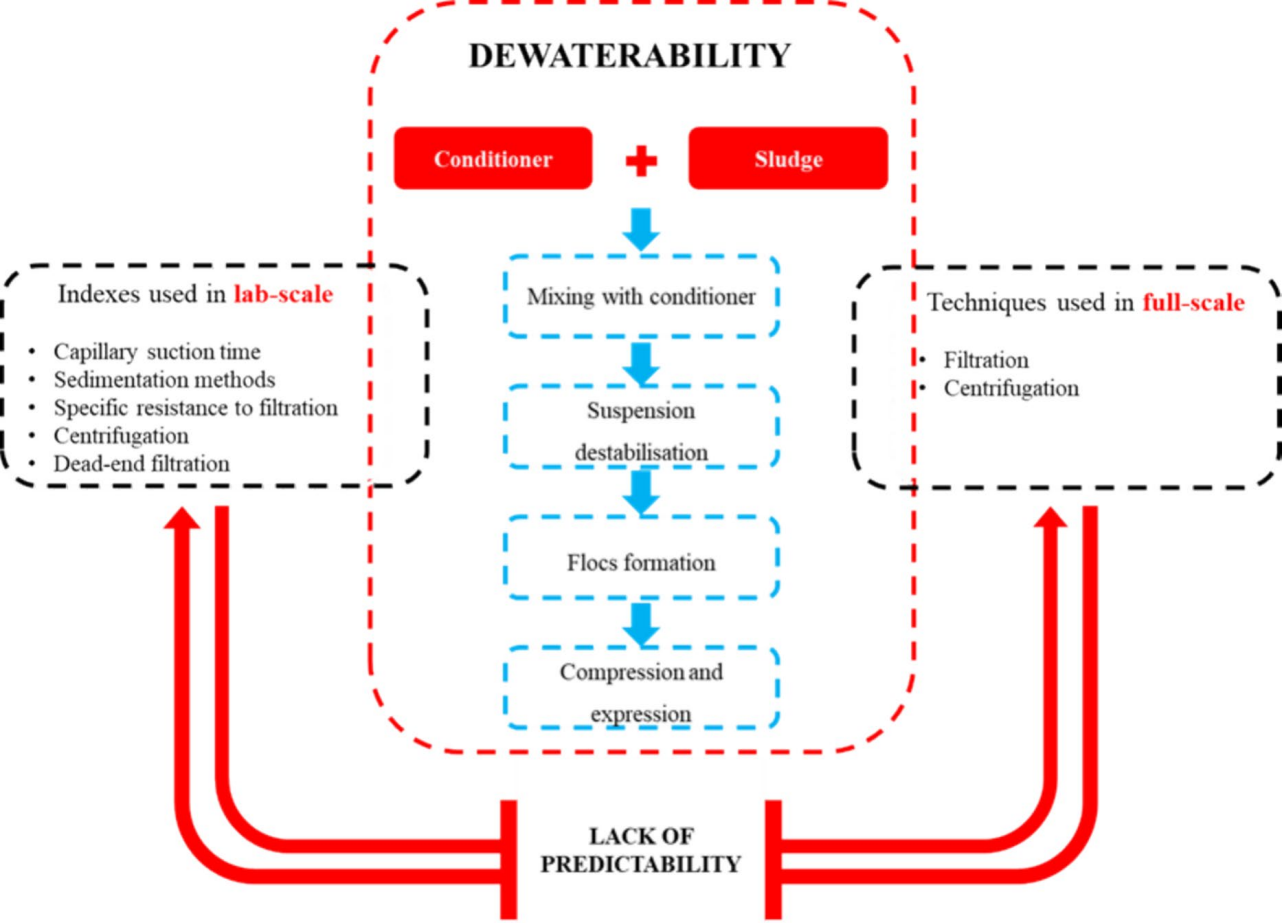

## Introduction

Dewatering of sewage sludge encompasses the process in which the volume of the sludge suspension is reduced due to the separation of a soluble effluent from a solid matrix. This process is preferably conducted mechanically, due to the lower cost of mechanical techniques compared with methods that involve state changes, such as evaporation. The capacity to remove water using mechanical methods from a sludge matrix is typically assessed by a parameter called dewaterability. In general, dewatering works as an umbrella term to describe processes that enhance solids concentration, reduce volume, and optimise the transportation of the final waste, such as filtration and centrifugation (Christensen and Dick 1985; Novak 2010; Christensen et al. 2015). Dewatering also increases the calorific power as a result of water removal, which reduces cost in places where incineration is used as the final disposal of the sewage sludge (Korving 2012; Politecnico di Milano 2019). Usually, the primary objective of dewatering is to reduce volume by increasing the solids concentration of sludge. However, depending on the context, it may also serve to harvest and reuse water. Besides sewage sludge treatment, dewatering methods find application in numerous contexts, such as sediments from dredging, industrial sludges resulting from precipitation-filtration processes, drinking water production, and pulp and paper processing (Chen et al. 2002; Scales 2006; Wojtanowicz 2008).

Regardless of the separation method employed, dewaterability is primarily dictated by the inherent characteristics of the suspension and the type of water contained in it. Bogue (1924), and later Heukelekian and Weisberg (1956) stated that not all water in the sludge interacts in the same way with the solid matrix, implying that there is non-extractable water when using mechanical methods. The notion of a theoretical dewaterability limit suggests that the ease of water removal varies based on the water-solids specific binding characteristics, defining free water and chemically bound water. Later, Catalano (1970) proposed a thermodynamic support for the found evidence, arguing that the bound water needs energy to break the binding with the solid matrix, and thus, it is more difficult to remove. The premise holds that mechanical techniques fail to extract bound water from the structure of solids. Consequently, the content of bound water can be regarded as a theoretical limit to attain.

Prediction of the final solids’ concentration and soluble effluent quality in dewatering processes requires the combination of several factors involving the sludge matrix, the dewatering process, and the conditioners used. Most industrial applications use conditioners to improve the dewatering process (Abu-Orf and Dentel 1997; STOWA 1998, 2023; DWA 2013). Conditioning operations physically modify the sludge structure, and such interactions define the final solids content of the solid fraction, and soluble effluent`s quality. Nonetheless, a comprehensive mechanistic framework explaining water retention and release in sludge flocs remains incomplete, particularly the molecular conformation at the water–solid interface (Wu et al. 2020).

Different standard empirical indexes can be found in the literature to predict full-scale dewatering behaviour. These predictors are based on measurements of particular characteristics of sludge (To et al. 2016). However, the dewatering process in full-scale operations encompasses multiple unit operations and phenomena, often oversimplified in laboratory tests, which has led to a lack of predictability in the field (Skinner et al. 2015). This lack of correspondence between laboratory predictions and full-scale behaviour hinders the upscaling of current dewaterability assessment methods, creating great challenges when predicting the effect of the application of new conditioners on novel and traditional matrices.

In our present review, we aim to set the phenomenological foundations involved in dewatering processes to analyse the capacity of laboratory assessments of dewaterability to predict the behaviour of full-scale dewatering processes. This will enable to evaluate the advantages and drawbacks of different assessment methods, while highlighting barriers that impede the comparison between lab-scale and full-scale results. We offer a compartmentalised perspective of the phenomena determining the dewatering of sewage sludge, suggesting four distinct phenomena that rule the dewatering process and must be considered when assessing dewaterability. Hopefully, this segmentation sets the ground to enable a separate comparison of each phenomenon in both laboratory and full-scale scenarios, improving the predictability of full-scale dewatering efficiency.

## Commonly used methods to dewater streams in sewage treatment

There are several methods to reduce the volume of suspensions in full-scale processes, such as vacuum filters, belt filter presses, centrifuges, and membrane filters (Chen et al. 2002). The main factors influencing the adoption of a specific technology comprise the volume of sludge to be ed, investment and operational costs, reuse of the extracted water, and conditioners price and availability. Nonetheless, within full-scale dewatering installations, the most used methods are filtration and centrifugation (Tunçal and Mujumdar 2023). For instance, in The Netherlands centrifuges, belt filters and filter presses comprise around 60% 23% and 17% of the installed capacity (Korving 2012).

Filtration provides solid–liquid separation based on particle size. Filtration ideally permits only the solvent and solutes (filtrate) to pass through the filtration medium, while the solid fraction is retained (Sutherland 2008; Tien 2012). The driving force behind filtration is a pressure difference existing between the two sides of the filtering medium, commonly known as transmembrane pressure (TMP) or ΔP. Additionally, specific filtration applications may employ supplementary driving forces such as osmotic pressure and electric fields to improve the process (Moldoveanu and David 2021). In general terms, filtration can be carried out in two primary modes: dead-end filtration, involving the creation of a cake over the filtration medium; and crossflow filtration, which provides back-transport mechanisms to physically prevent cake formation (Van der Bruggen 2018). Despite the availability of crossflow filtration, it is hardly utilised in high solids dewatering processes, with (semi)dead-end filtration being the predominant technique, due to the high viscosity of the matrices being dewatered. Within dead-end filtration methods, belt (although not fully dead-end filtration due to the continuous operation) and press filters are commonly encountered in full-scale installations, which can reach solids contents as high as 15–20% (Dick et al. 1980; Sparks 2012). Chamber filter presses can also be used, where mechanical/hydraulic pressure is applied to create the TMP, which can reach up to 16 bar. Belt filters normally work in three stages: i) gravity drainage; ii) compression; iii) shear presses. They can operate in continuous mode, only stopping the process to do maintenance.

Moreover, centrifugation achieves solid–liquid separation based on a density difference. During centrifugation, a centrifugal force is applied, which surpasses the gravitational force, accelerating the solids-liquid separation. As a result, settling velocity of particles is artificially enhanced, reducing the period for settling (Huang et al. 2014). A wide variety of centrifugal dewatering equipment is used in industry, but for full-scale sewage sludge dewatering applications, solid bowl machines (also known as decanter centrifuges), are commonly used. Solid bowl centrifuges also include a scroll conveyor, a cast iron case, and a main drive (Novak 2006). The main drive rotates the bowl, and the back drive controls the conveyor speed. Centrifuges are offered in two configurations: co-current i.e., cake and centrate travel in the same direction, and counter-current i.e., cake and centrate travel in opposite directions. To modify the operational parameters, the operators can vary the feed rate, conditioners, dosage rate, and bowl conveyor speeds.

## Use of coagulants and flocculants as the main chemical conditioning strategy to reach high solids content

Conditioning of sludge is a widely adopted practice used to prepare the biosolids for subsequent solid–liquid separation processes. Conditioning can be understood as a series of physical, chemical, or biological techniques intended to change the sludge structure to facilitate solids removal and/or to improve soluble effluent quality (Cornier et al. 1983; Senthilnathan and Sigler 1993; Dentel 2001).

The most used chemical conditioning technique is the addition of coagulants and flocculants. They are used in different steps of the sewage treatment, such as primary and secondary clarification, as well as during sewage sludge dewatering (Teh et al. 2016; Abujazar et al. 2022). Tchobanoglus et al. (2003) define coagulation as “the process of destabilising colloidal particles so that particle growth can occur as a result of particle collisions”. The same book defines flocculation as “a transport step that brings about the collisions between the destabilised particles needed to form larger particles that can be removed readily by settling or filtration”. Despite the differences existing between the phenomena of particle destabilisation (coagulation) and the growth of the destabilised particles (flocculation), the concepts of coagulation and flocculation have become interchangeable in the literature (Semerjian and Ayoub 2003; Verma et al. 2012; Lee et al. 2012; Wei et al. 2018; Zhao et al. 2021; Kurniawan et al. 2022). The difficulty in differentiating between both phenomena grounds on the capacity of coagulants and flocculants to produce the phenomena of coagulation and flocculation interchangeably. In our current article, the definition proposed by Adachi (1995) will be adopted, wherein coagulants encompass low molecular weight salts, and flocculants involve the use of polymers, also often called polyelectrolytes. The definition based on the conditioner and not the phenomena assumes that both coagulation and flocculation can simultaneously occur, using both coagulants and flocculants.

Most industrial-level coagulants consist of salts based on polyvalent cations such as Fe^3+^ or Al^3+^ (Licskó 1997). These salts are extensively utilised in solids separation due to their cost-effectiveness and their ability to cause charge neutralisation. Additionally, their insoluble amorphous hydroxides induce sweep flocculation (Duan and Gregory 2003; Thakur et al. 2021). Similarly, polymeric inorganic salts like polyaluminium chloride, poly-alumino-iron-sulphate, and poly ferric sulphate serve the same purposes (Jiang and Graham 1998; Delgado et al. 2003; Zouboulis et al. 2008; Kushwaha et al. 2010). However, the use of metal-based coagulants has been a topic of controversy due to concerns related to alkalinity consumption, and potential adverse effects on living organisms when they remain in the supernatant, particularly in the case of Al, which has been linked to toxic effects and Azhaime disease (Martyn et al. 1989; Flaten 2001; Becker and Asch 2005; Al-Mutairi 2006; Theodoro et al. 2013; Zaid et al. 2020). Inorganic coagulants have exhibited some drawbacks in industrial use, such as requiring a high quantity to achieve separation, pH-dependent performance, and difficulties in separating small particles (Sharma et al. 2006). Due to these reasons, inorganic coagulants have fallen into disuse in processes that require high solids content, or when used nearly always in combination with organic flocculants.

Organic flocculants comprise water-soluble, high-molecular-weight polymers derived from acrylamide, acrylic acid, diallyl dimethyl ammonium chloride, styrene, sulphonic acid, among other monomers (McGuire et al. 2006; Bolto and Gregory 2007; Wang et al. 2019). Organic flocculants are the predominant type of conditioners used in industrial applications because of their conformational flexibility during the manufacturing process, cost-effectiveness, and high performance. The key parameters used to characterise organic flocculants include charge density, molecular weight, and polymeric structure (linear, branched, or net). Depending on the nature of the suspension being dewatered, a variety of flocculants, including cationic, anionic, non-ionic, ampholytic, or a combination, can be used (McGuire et al. 2006; Koshani et al. 2020). Typically, cationic flocculants are used in sewage treatment to neutralise negatively charged suspensions, such as microorganisms (Mortimer 1991; Ubbink and Schär-Zammaretti 2007). They are also used in other applications, such as sanitary landfill leachate, natural organic matter in surface water, sewage sludge, struvite, wastewater influent, among others (Tatsi et al. 2003; Ginos et al. 2006; Jarvis et al. 2006; Le Corre et al. 2007; Pambou et al. 2016; Kooijman et al. 2017). Conversely, anionic flocculants are predominantly employed to aggregate positively charged suspensions, such as hematite wastewater, fibre cement, kaolinite, methylene (Caskey and Primus 1986; Taylor et al. 2002; Negro et al. 2005; Ng et al. 2015; Sadangi et al. 2020; Feng et al. 2021; Guan et al. 2021). Although rarely employed with high solids contents, combinations of cationic coagulants, either in tandem with charged or neutral flocculants, can be applied to achieve charge neutralisation through the coagulant and bridging/floc formation through the flocculant structure (Pearse et al. 2001; Ahmad et al. 2008; Zhao et al. 2012; López-Maldonado et al. 2014; Yang et al. 2019; Wang et al. 2023). The flocculant’s elevated molecular weight indicates that the organic polyelectrolyte will have an increased hydrodynamic radius when in solution (St Thomas et al. 2021), enhancing its bridging capacities. Moreover, a higher molecular weight also implies that when in an aqueous solution, the flocculant will elevate the viscosity of the solution (François et al. 1979). High-viscosity solutions may pose problems when pumping the conditioner to be mixed with the sludge. Large differences in the viscosity of the conditioning liquid and the sludge viscosity may lead to mixing problems. For this reason, the polyelectrolyte is typically dosed via diluted solutions with 0.1–2%DS of active polymer content.

Despite the widespread adoption of coagulation/flocculation, there is limited available information that can help to predict the suitability and dosage of coagulants/flocculants for specific applications and equipment, or to establish correlations with the chemical structure of the matrix being dewatered (Bolto and Gregory 2007). Consequently, in full-scale installations, the effectiveness of the utilised conditioners is typically determined through trial and error. Trial and error heavily relies on the experience of the operators and cannot be extrapolated among different types of dewatering machines. For example, flocculants for centrifugation are more “structured”, whereas those for filter presses have a more linear structure. Under this context of lack of mechanistic models, the tests outcomes are impacted by the indexes used to assess dewaterability. A proper protocol for assessing the use of conditioners should replicate the conditions under which the suspension will undergo dewatering, considering all the phenomena encountered during the process to avoid trial-and-error biases.

## Proposed phenomena occurring during dewatering of sewage sludge

**Fig. 1:**
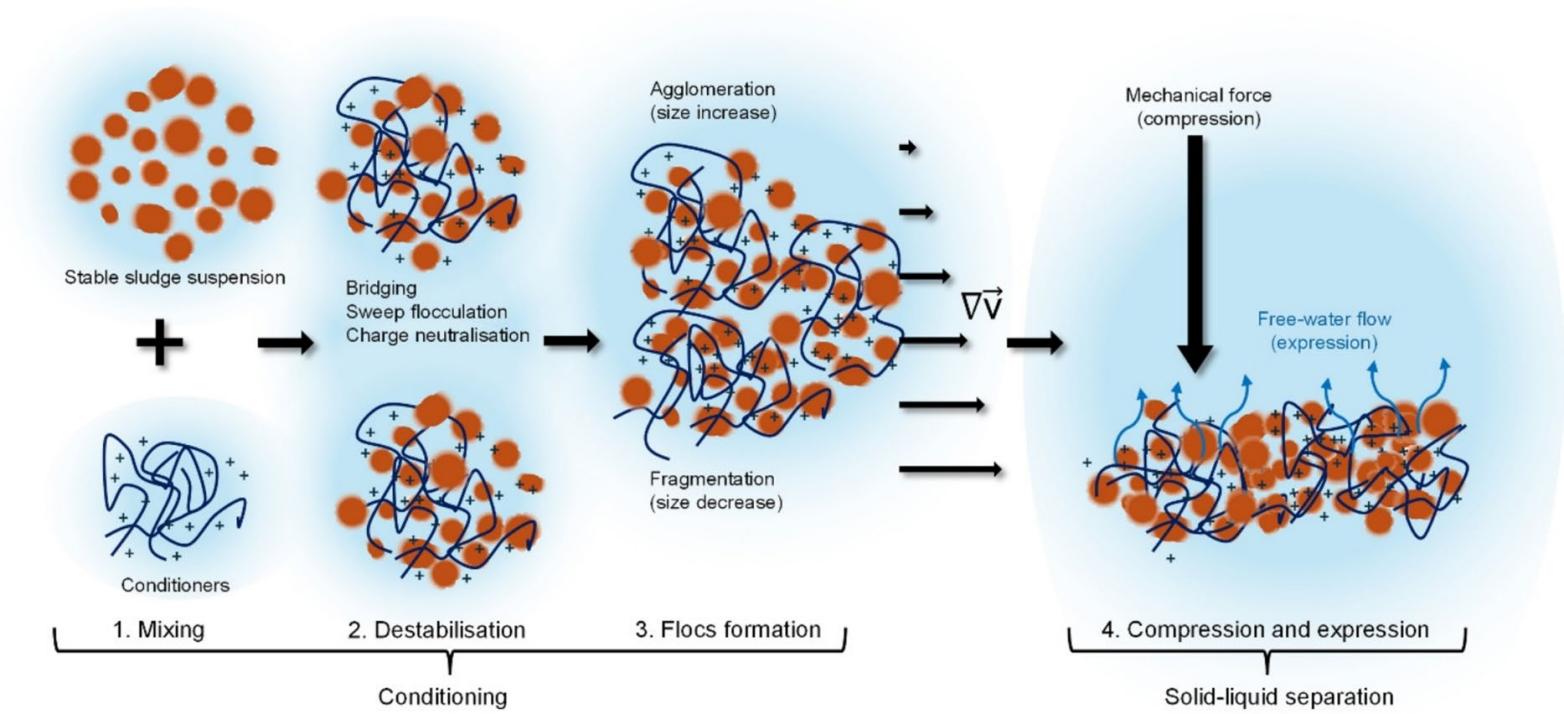
Phenomena comprising dewatering, considering a negative suspension surface charge (not shown for simplicity), and positively charged chemical conditioners. $\nabla \mathop{v}\limits^{\rightharpoonup}$ represents the velocity gradient in the liquid

This section scrutinises the dewatering process, defining phenomena that are common to all dewatering processes, regardless the equipment used. The four phenomena proposed comprise mixing, suspension destabilisation, flocs formation, and compression and expression. In practice, some of these phenomena may take place simultaneously. However, it is useful to examine them separately for a better understanding of the underlying mechanisms. These four phenomena are proposed based on the assumption that full-scale processes mostly use chemical conditioners (coagulants and flocculants), to improve the dewaterability of sludge. Figure 1 shows a diagram of the four proposed phenomena occurring during the dewatering process.

### Mixing of sludge suspension and conditioner

The mixing process seeks to facilitate the interaction between the sludge and the conditioners. Ideally, complete homogeneity during mixing ensures that the conditioner is uniformly distributed among the solids in the sludge, leading to the destabilisation of the sludge suspension. Fluid dynamics of the mixing of sludge and conditioners need an understanding of the rheological characteristics of both fluids and the mix, although this is rarely discussed in the literature.

To ensure the even dispersion of conditioner molecules into the sludge suspension, they must be transported from the highly viscous matrix in the polymer stock solution to the sludge particles. The design of the mixing process heavily relies on the rheological characteristics of the sludge and polymer. In broad terms, sludge and polymer solutions exhibit non-Newtonian shear-thinning behaviour, e.g., their viscosity decreases with increasing shear rate (Kulicke et al. 1982; Lewandowska 2007; Bjrn et al. 2012; Hong et al. 2017; Hii et al. 2019). Nonetheless, the reduction in viscosity of sludge and polymeric conditioners during high shear rates can be advantageous for the mixing process. Therefore, the rheological behaviour of sludge and conditioners at specific mixing conditions determines the mixing performance. Moreover, the sludge viscosity and density are determined by the solid's concentration of the sludge suspension and the type of sludge. The density of the solids in the sludge is higher than that of water, resulting in a higher than water density at elevated solids concentrations (Vincent et al. 2011; van den Berg et al. 2023). In the same line, the viscosity of the sludge increases progressively with the increased solids concentrations (Lotito et al. 1997; Eshtiaghi et al. 2013; Markis et al. 2014; Cheng and Li 2015). Similarly with the sludge, the density and viscosity of the conditioners depend on the concentration of the polymer solution prepared and operation conditions for the dewatering process, such as shear rate, and polymer type (Saveyn et al. 2008, 2010).

The concentration at which the conditioner is commercialised is typically predetermined by the manufacturer. However, the optimal concentration at which the polymeric solution is prepared to be mixed with the sludge has not been extensively addressed in the literature. Determining the proper mixing concentration must consider viscosity, density, and the storage time of the final solution, to prevent degradation over time due to hydrolysis. Concentration is relevant since it has been found that degradation occurs faster for more diluted solutions, which can be prevented by acidifying the polymers (Saveyn et al. 2008, 2010). However, a highly concentrated solution may challenge pumping and mixing, while a low concentration solution may be voluminous and could degrade if not promptly used.

Various parameters can be used to characterise the mixing operation, such as mixing time, circulation time, minimum agitation speed, and mixing power (Villermaux 2019). Nonetheless, the comparison among different technologies is facilitated using comparable parameters. The product G·t was proposed by Novak (2006) to compare different shear-induced equipment. In this parameter, G represents the mean velocity gradient (1/s), and t is the shear time (s). Noteworthy, the conditioning dose has been found to depend on G·t (Lynch and Novak 1991; Murthy et al. 2004), which supports the use of G·t to compare mixing phenomena.

### Sludge suspension destabilisation

It is intrinsically understood that stable sludge suspensions subjected to dewatering are impacted by Brownian motion to a certain extent, indicating that the particles within the suspension do not easily settle in the presence of external forces. The destabilisation process reduces the impact of Brownian motion upon the particles in the suspension, destabilising them and promoting flocs formation (Larsson et al. 2012). Destabilisation is presumed to occur just after and during the mixing of the sludge with the conditioner. Destabilisation is achieved through the process of charge neutralisation, in which the repulsive forces of the colloids' double layer are counteracted by the addition of opposite charges (Matilainen et al. 2010). Once the suspension is destabilised, the particles no longer repel each other and can form larger aggregates (flocs).

Destabilisation of the suspension can be accomplished using various conditioners that induce charge neutralisation in addition to bridging among the forming and formed flocs (Gill and Herrington 1987). Adequate dosage and type of conditioner have proven to be key parameters for suspension destabilisation. A dosage below the optimal level may prove insufficient to achieve the desired effect, while an excessive amount could be detrimental to destabilisation and soluble effluent quality, due to charge reversal (Beltrán Heredia and Sánchez Martín 2009; Hyrycz et al. 2022). Typically, during the coagulation of sewage sludge, cations and/or positively charged polymers are added to the matrices to balance the negatively charged EPS (Ramesh et al. 2006; Bala Subramanian et al. 2010). In this regard, trivalent cations, such as Fe^3+^ and Al^3+^, are commonly used for charge neutralisation of negatively charged particles (Duan and Gregory 2003). Additionally, organic polymers are employed, and beyond charge neutralisation, they also interact by bridging and patching the colloid surfaces, promoting flocculation (Lee et al. 2014). In addition to the used conditioner, divalent cation bridging (DCB) theory helps to understand flocculation (Spicer and Pratsinis 1996; Sobeck and Higgins 2002). According to DCB theory divalent cations bridge negatively charged moieties in the EPS matrix of sludge, promoting aggregation and stabilisation of the EPS matrix and thus enhancing bio-flocculation (Sobeck and Higgins 2002). Under DCB theory, Ca^2+^ and Mg^2+^ play a fundamental role in the dewatering of suspensions since these are the main divalent cations ubiquitously present in the sludge being dewatered.

### Flocs formation

This phenomenon corresponds to the aggregation of the already mixed and destabilised particles. The aggregation increases the size of the particles to form flocs. The ultimate size of the flocs is influenced by both, the inherent characteristics of the sludge and the hydrodynamic forces exerted in the bulk of the liquid (Spicer and Pratsinis 1996). Noteworthily, the use of conditioners contributes to enlarging the floc size and consequently increasing their settling velocity (Sieliechi et al. 2016). During flocs formation, it is crucial to minimise shear forces to prevent the shear-induced breakup of the formed flocs (Mikkelsen and Keiding 1999; McCurdy et al. 2004). Consequently, the final size of the flocs represents a delicate dynamic equilibrium between two mechanisms: aggregation and shear-induced fragmentation (McCurdy et al. 2004). The shear effects are often overlooked in discussions regarding the use of full-scale equipment such as centrifuges, in which shear effects are certainly present.

Mainly four flocs aggregation mechanisms are recognised in coagulation-flocculation processes: charge neutralisation, bridging, patch flocculation, and "sweep” flocculation (Packham 1965; Snodgrass et al. 1984; Bache et al. 1997; Swenson et al. 1998; Biggs et al. 2000; Ghernaout and Ghernaout 2012; Hyrycz et al. 2022). On the other hand, fragmentation may occur due to the erosion of solids or collisions with other solids. Several models have been proposed to comprehend the growth and breakup process based on solids concentration and shear rate in the suspension (Haugaard Mikkelsen and Keiding 1999; Mikkelsen 2002). Consequently, depending on the applied conditions, growth and breakup processes can be reversible phenomena in activated sludge (Chaignon et al. 2002). Most of the aggregation-fragmentation models rely on the Smoluchowski equation (Smoluchowski 1918), which describes how the size of aggregating particles changes over time due to collisions. Also, modifications of Smoluchowski equation have been proposed to include effects such as DLVO forces and hydrodynamic interactions (Thomas et al. 1999).

### Compression and expression

After the formed flocs are large enough to be efficiently separated from the bulk, an external driving force such as gravitational, pressure, or centrifugal force must be applied to reach the solid–liquid separation (Shah et al. 2020). The solid–liquid separation in which the water is withdrawn and thus the sludge reduces its volume is called compression. During the compression phase, particles undergo deformation, causing water to be squeezed both from within individual sludge particles and between them (Novak 2006; Peeters et al. 2011b). In practical terms, once the floc structure is established and the flocs have gained a considerable size, they are compressed together into existing water voids, allowing free water to flow through the channels surrounding the flocs structure (Yusa 1984). Given that the density of solids in the sludge is typically higher than that of water, density increases during this step.

An expression phase follows the compression stage, characterised by sustained pressure application after permeate pressure falls and the water voids are no longer filled, keeping a constant size (Tiller et al. 1987). While compression by such removes some water, it is insufficient for producing dewatered cakes with the commonly desired solids concentrations, and full-scale dewatering heavily relies on the expression phase. During the compression and expression phase, the ultimate compression of sludge depends on the stress–strain characteristics of the sludge. Besides, the final solids content of the sludge is influenced by the speed at which the sludge is compacted and expressed. Lower speeds yield higher solids contents (Chaari et al. 2003). Additionally, when the load is no longer applied, the sludge will revert to its original shape based on the compressibility/expansibility of the material (Phanikumar and Raju 2020) and depending on the machine design this may lead to sucking up of water or air. Therefore, this re-bounce effect will also contribute to the final solids content of the sludge (Schwartzberg 1997).

## Factors determining dewatering

There is neither a unique parameter that defines dewaterability nor certainties of which matrices characteristics greatly influence the dewatering process. Consequently, several characteristics of the sludge matrix have been identified to influence the capacity to remove water from the sludge matrices in the literature. This lack of consensus obscures the identification of meaningful parameters that allow the definition of mechanistic models that predict the dewatering behaviour under different scenarios. Table 1 shows the variety of parameters in the sludge matrices that have been found to impact the dewaterability of sludges.

**Table 1:** Factors that can impact dewaterability

Characteristic influencing the dewaterability	Effect(s)	References
Conditioner type and dose	Improvement of dewaterability when applied in the proper amount	Tenney and Cole (1968), Christensen et al. (1993), Novak et al. (1999), Cao et al. (2016)
Rheology	There is relationship in with certain rheological parameters in particular conditions such as viscosity, compressibility, storage and loss moduli. However, this is complex, depends on sludge characteristics and conditioning processes and cannot be broadened to different scenarios	Hou and Li (2003), Stickland (2015), Yeneneh et al. (2016), Wang et al. (2017), Jiang and Zhou (2020)
Sludge’s high compressibility	Higher compressibility renders worse dewaterable sludge	Novak et al. (1999), Stickland (2015)
EPS structure	Positive correlation with specific resistance to filtration (SRF)	Hankermeyer and Tjeerdema (1999), Li and Yang (2007), Xiao et al. (2016)
Surface charge expressed as streaming current and zeta potential (ZP)	Streaming current and ZP close to zero enhances dewaterability	Walker et al. (1996), Abu‐Orf and Dentel (1998), Dentel et al. (2000), Braguglia et al. (2006), Guan et al. (2012)
Orthophosphate	Elevated orthophosphate concentrations worsen dewaterability	Bergmans et al. (2014), Alm et al. (2016), Ghodsi et al. (2021)
Ratio’s between mono-, di and trivalent cations	Elevated concentrations of divalent cations help dewaterability	Higgins and Novak (1997), Biggs et al. (2001), Sobeck and Higgins (2002), Jin et al. (2004), Higgins et al. (2004a, b), Peeters et al. (2011a), Alm et al. (2016), Liu et al. (2021)
Particle size and presence of colloids	Big particles tend to enhance dewaterability. Colloids in general worsen dewaterability	Karr and Keinath (1978), Shao et al. (2009), Novak (2010)

Table 1 shows a diversity of parameters influencing dewaterability reported in the literature, which reflects the complex nature of sludge matrices and the multiple mechanisms governing water retention and release during dewatering. However, the large number of studies focusing on different individual parameters, each reporting correlations with dewaterability, also highlights the absence of a clear consensus regarding which factors exert the dominant control over the process. Rather than converging toward a unified set of governing variables, the literature presents a fragmented view in which different sludge characteristics influence dewaterability. This diversity of proposed parameters suggests that dewaterability cannot be reliably described by a single sludge property and reinforces the need for a more integrated mechanistic framework capable of linking sludge composition, structure, and conditioning processes with dewatering performance. Considering the complexity of isolating the parameters that influence sludge dewaterability and thus, the dewatering process, dewaterability has been typically assessed using empirical indexes. Empirical indexes commonly base the assessment in a parameter that can be effortlessly measured in lab and full-scale.

## Commonly used indexes to assess dewaterability

Indexes describe a property of the sludge matrix that resembles the effect of the phenomena shown in Fig. 1. An ideal index to assess dewaterability needs to be easy to implement, resemble the full-scale results, and provide meaningful comparable parameters. Several standard assays have been used to assess dewaterability, which rely on different readily measurable parameters. These assays use several driving forces such as centrifugal force, TMP, or capillary force to reach the solid–liquid separation. This section describes commonly used indexes to assess dewaterability.

### Capillary suction time (CST)

CST stands as maybe the most widely employed index to assess dewaterability in both laboratory and practice (Gray 2015). It is claimed to serve as a standard for evaluating the efficacy of coagulants, flocculants, or conditioners, due to its straightforward implementation and operation. CST is often used after adding coagulants or flocculants to the sludge to determine the optimal dosage. However, the conditioning often leads to very short suction times due to a large free water excess, leading to high variability in the results. Alternatively, the CST test can also be used as a tool to characterise the sludge without polymer addition, which gives information on changes in sludge properties.

The CST procedure involves confining a suspension in a column, positioned on a porous layer, often a filter paper. The filter paper facilitates the radial flow of free water from the suspension column due to the capillary pressure exerted by the filtering medium. This capillary pressure complements the hydrostatic pressure from the column positioned on the filtering medium, expediting the process (Meeten and Smeulders 1995). CST measures the time it takes for the permeate to expand the wet radius on a filtering medium from one point to another positioned at a greater distance from the suspension column, and therefore in indicates the speed at which the water is extracted from the sludge interstices. The wet radius is frequently measured by electronically sensing the change in the conductivity of the filtering material. Standardised and patented apparatuses, with pre-established dimensions, are typically used for CST measurements.

Some efforts have been made to correlate CST measurements with other dewaterability indicators, with different levels of success (Lee and Hsu 1994; Mikkelsen 2002; Scholz 2005; Sawalha and Scholz 2010). Operational factors influence CST, such as filtration paper, temperature, solids concentration, and instrument geometry (Gray 2015). In general, if the aforementioned parameters remain constant, dewaterability can be assessed in samples from the same origin, revealing relative changes in the same sludge matrix. However, it provides limited capability to compare samples from different origins or to use different conditioners during the assessment.

### Sedimentation methods

Sedimentation methods are mostly used in diluted suspensions, which are transparent, and the sedimentation can be visually observed. For dewatering purposes, it requires dilution, and this can influence the analysed sludge and thus the outcome of the measurement. One of the most used parameters to assess the settleability of biosolids is the sludge volume index (SVI) (Ramalho 2013). SVI has been the standard method to calculate the settling properties of sludge and consists of a 30-min settlement experiment, followed by a visual observation of the settled bed. SVI is calculated using Eq. 1. Due to the standardisation of the technique, the samples can be fairly compared with the literature to assess the sludge settling quality.

1 $${\text{SVI(ml/g) = 1000}}\left( {\frac{{{\mathrm{mg}}}}{{\mathrm{g}}}} \right)\frac{{{\text{settled sludge volume }}\left( {\frac{{{\mathrm{ml}}}}{{\mathrm{L}}}} \right)}}{{{\text{mixed liquor suspended solids }}\left( {\frac{{{\mathrm{mg}}}}{{\mathrm{L}}}} \right)}},$$

Another sedimentation parameter used in lab-scale is zone settling velocity (ZSV). Although less used than SVI, ZSV is still used to estimate the sedimentation velocity. ZSV describes the maximum velocity at which the level of the sludge bed lowers in a sedimentation curve versus time (Association et al. 1917).

### Centrifugation methods

Centrifugation employs the same working principle as sedimentation, using a density difference to reach separation. During centrifugation, a driving force (centrifugal force) greater than gravity is used to expedite the process. In lab-scale applications, it has proven to be a fast and simple method to assess dewaterability (Usher et al. 2013). When comparing centrifugation across various devices, a common parameter employed to measure the force intensity is the relative centrifugal force (RCF). RCF represents the centrifugal force with respect to the gravitational acceleration being comparable regardless of the device geometry. RCF is determined using Eq. 2.

2 $${\text{RCF = }}\left( {\frac{{\left( {\frac{{{2} \cdot \pi }}{{{60}}}} \right)^{{2}} }}{{{100}\frac{{{\mathrm{cm}}}}{{\mathrm{m}}} \cdot {9}{\text{.80665 }}\left( {\frac{{\mathrm{m}}}{{{\mathrm{s}}^{{2}} }}} \right)}}} \right) \cdot {\text{ radius (cm)}} \cdot {\mathrm{RPM}}^{{2}} { = 1}{\text{.118 }} \cdot {10}^{{ - {5}}} \cdot {\mathrm{radius}}\,{\mathrm{(cm)}} \cdot {\mathrm{RPM}}^{{2}} ,$$

Centrifugation is an interesting option to assess dewaterability since RCFs are comparable in different devices used to measure dewaterability. However, it is essential to address the quality of the centrate, to ensure that the suspended colloidal material is removed in the same manner as in industrial applications.

Moreover, Higgins et al. (2014) introduced a modified lab-scale centrifugal technique, which combines elements of both filtration and centrifugation. The concept involves dividing a centrifuge into two chambers by installing a perpendicular filtering cloth and a central support. This setup facilitates dead-end filtration using the centrifugal forces generated by the rotating sample. In this configuration, RCF serves as an indicator of the dewatering technique's intensity. Additionally, a multiplication of time and rotational acceleration, referred to as "RCF·t," is considered comparable parameter, which considers both filtration intensity and duration. As well as with the filtration methods, the centrifugation-filtration method allows to assess of the four previously mentioned phenomena, and the application of conditioners, which may help to resemble the full-scale data in a lab-scale.

### Specific resistance to filtration (SRF)

The SRF is grounded in Darcy's law, which indicates that TMP is equivalent to the head loss of the fluid passing through the porous filtering cake (Agerbæk and Keiding 1993). Using this approach, the permeate flow rate relies on the permeate viscosity, filtering medium dimensions, and the resistance posed by the cake and filter to filtration. If the permeate flow and filtering medium dimensions are known, the permeate flow rate through the cake indicates the cake's resistance. To determine the SRF, Eq. 3 is used. The calculation of SRF involves utilising the slope “b” from a graph of t/V versus V, as expressed in Eq. 4.

3 $$\frac{{\mathrm{t}}}{{\mathrm{V}}}{ = }\left( {\frac{{\eta \cdot {\mathrm{SRF}} \cdot {\mathrm{c}}}}{{{2} \cdot {\mathrm{TMP}} \cdot {\mathrm{A}}^{{2}} }}} \right){\text{V + }}\frac{{{\upeta } \cdot {\mathrm{R}}_{{\mathrm{m}}} }}{{{\mathrm{TMP}} \cdot {\mathrm{A}}}}{\text{ = b}} \cdot {\text{V + }}\frac{{\eta \cdot {\mathrm{R}}_{{\mathrm{m}}} }}{{{\mathrm{TMP}} \cdot {\mathrm{A}}}},$$

4 $${\text{SRF = }}\frac{{{2} \cdot {\mathrm{b}} \cdot {\mathrm{TMP}} \cdot {\mathrm{A}}^{{2}} }}{{\eta \cdot {\mathrm{c}}}},$$

where: V = permeate volume, c = solids concentration in the suspension, A = filtration area, R_m_ = membrane resistance, η = dynamic viscosity of the permeate.

Noteworthy, membrane resistance is unnecessary to calculate the SRF. Nevertheless, it is imperative that the membrane employed in lab-scale experiments closely resembles the filtering cloth used in full-scale processes. Using different filtering media may affect the permeate quality and cake porosity and thus impact the SRF. The main comparable parameter in dead-end filtration at lab and full-scale is the nominal pore size. A smaller nominal pore size in lab-scale dewaterability assessments may result in a higher SRF compared to full-scale results since finer particles are retained in the cake. Additionally, when evaluating SRF, careful control of the TMP is essential, and accurate measurements of "c" (solids concentration) and "η" (permeate’s viscosity) are required. In most practical scenarios, the permeate’s viscosity is similar to the one of water, which closely resembles a Newtonian fluid, allowing to measure "η" with routine viscometers. The literature commonly suggests a TMP of 50.7 kPa (0.5 atm) for SRF measurements (Christensen and Dick 1985). However, a TMP of 50.7 kPa may not be sufficient to reach expression of compressible sludges since the pressure is commonly several times larger in filtration devices (Stickland et al. 2005; Lee et al. 2007; Skinner et al. 2015).

### Other dead-end filtration methods

Filter presses stand as cost-effective laboratory equipment, enabling the assessment of dead-end filtration techniques under conditions similar to full-scale filters. Widely employed in the food and beverage, chemical, pharmaceutical, and environmental sectors, these presses typically consist of a piston for compressing the suspension, a pump generating the TMP, and a reservoir for collecting the permeate (Ruiz et al. 2010; Guerrini et al. 2015). Conversely to SRF apparatuses, which commonly work with vacuum, these presses use positive pressure to achieve a TMP higher than atmospheric. The use of positive pressure allows more flexibility than vacuum use, which limits maximum TMP to a pressure in the range of the atmospheric pressure. Moreover, most devices enable the monitoring and control of TMP, which allows them to resemble full-scale conditions. Also, the filtration cloth used in such equipment can closely resemble that utilised at full scale, which is crucial to compare results (Raman and Klima 2021). Another notable advantage of these devices is their capability to assess permeate and cake qualities upon completing a run, allowing for the comprehensive evaluation of all four phenomena formerly defined in the dewatering process. Nevertheless, sludge´s high compressibility may hinder dewaterability assessment in matrixes with different solids concentrations, which often show varying results depending on the cake thickness. Nonetheless, in the same way as centrifugation methods, dead-end filtration methods allow for assessing the all the proposed phenomena during dewaterability, which may help to resemble the full-scale data in a lab-scale.

### Novel indexes proposed in the literature

In addition to the traditionally used assessment indexes, some novel methods have been proposed, mainly at research level. Drainage-based indexes represent a practical approach that directly measures water release rates under gravity, providing simple approaches which have the potential to be e widely adopted. For instance, drainage index (DI), proposed by Olivier et al. (2018), has demonstrated predictive capability for gravity-driven dewatering equipment. Rheological parameters, particularly compressive yield stress has also emerged as a predictor of dewatering performance. Liu et al. (2024) demonstrated that compressive yield stress correlates with final cake moisture content across various conditioning methods. These parameters have shown to be particularly valuable for pressure-driven dewatering technologies such as plate-and-frame filters and filter presses. Moreover, image analysis and moisture content estimation methods have emerged to position computer vision and machine learning as tools to provide real-time, non-invasive dewaterability assessment. Rumahorbo et al. (2025) developed regression models that estimate cake moisture content from digital images, enabling automated process control. This approach offers significant advantages for continuous and non-invasive monitoring or real-time applications. In addition, bound water and water distribution measurements have been developed employing techniques such as differential scanning calorimetry, nuclear magnetic resonance, and thermogravimetric analysis, aiming to quantify different water fractions within sludge matrices (Lee and Lee 1995; Mao et al. 2016; Zhang et al. 2019). Also, fractal dimension and structural parameters have been used to quantify the geometric complexity and porosity of sludge flocs and aggregates. Sun et al. (2015) showed that fractal dimension correlates with dewaterability, with higher values indicating more compact, less permeable structures that resist water removal. These parameters provide fundamental insights into how conditioning agents may modify sludge microstructure.

## General considerations when using dewaterability indexes

Despite the models present in the literature (McCabe et al. 1993; Huisman and van Kesteren 1998; Yukseler et al. 2007), methodological biases make dewaterability and thus dewatering process’ prediction of results prone to inaccuracies (Sawalha and Scholz 2010; To et al. 2016). As a result, dewaterability is typically estimated using the formerly described indexes, which, in our understanding, do not comprehensively address all the phenomena experienced by the sludge and conditioners during the dewatering process. Since not all the phenomena are considered during the dewaterability assessment methods, the results can be unreliable and lack predictability. This section presents the main considerations when assessing dewaterability using the formerly explained indexes, and the phenomena involved during the assessment. The phenomena of mixing, suspension destabilisation, and flocs formation are common to all the indexes analysed. Nonetheless, compression and expression show evident differences and are analysed separately.

### Mixing

Homogeneity in mixing is generally achieved when the fluids spend sufficient time under adequate shear stress in a homogenisation chamber or a pipeline. Therefore, to achieve proper homogenisation, sludge and conditioner interaction needs to be facilitated. In full-scale installations, a dedicated mixing process involves intricate fluid mechanics and a careful selection of appropriate equipment. The mixing of the conditioner and sludge can be carried out in a designated mixing chamber or by directly introducing the conditioner into the pipeline through which the sludge is conveyed to the dewatering device. In-line mixing, also known as axial mixing, involves three mechanisms: diffusion, eddy convection, and bulk convection. Due to the intricacy of the phases involved, estimating axial mixing based on fundamental principles is challenging (Ekambara and Joshi 2003).

In lab-scale, mixing time and intensity need to be determined in preliminary tests, regardless of the technique used to assess dewaterability. Consequently, it would be a good practice to register and report the mixing time and equipment during dewaterability assessment. As discussed, the mixing intensity can be assessed using “G·t” coefficient, which may allow the comparison of different configurations (Higgins et al. 2014). Moreover, mixing intensity needs to be adjusted to avoid the degradation of polymeric conditioners. Proper mixing in lab scale may increase the sample volume needed to assess dewaterability, particularly in tests such as CST or SRF, which commonly use a limited amount of sample.

Considering that mixing heavily relies on the rheology of the suspensions, conditioners pre-dilution using the same water quality as in full-scale is advised since some parameters, such as conductivity, can modify the conditioners' properties. (Chimamkpam et al. 2011; Boshrouyeh Ghandashtani et al. 2022). Moreover, Quezada et al. (2018) found using modelling that increased salinity (NaCl 0.006–0.6 M) favoured the conditioner adsorption, which also implied a reduction in its solution dimensions, thus limiting bridging capacity. However, the diversity of conditioning agents and sludge matrixes demands experimental research focused on the particular application. Finally, it is also advised to ensure that the concentrations at which the polymers and sludge are mixed are the same in all the tests being compared to simulate the rheological behaviour during mixing.

### Suspension destabilisation

The main factor in controlling the suspension stability is the conditioner-to-sludge ratio (conditioner dose). Moreover, to improve the reliability of the dewatering assessment methods, it is evidently recommended to use the same conditioner in lab-scale tests as the one used in the full-scale process trying to mimic. Also, to compare among different conditioners, the best option would be to use the grams of active polymer per gram of conditioner as a base for comparison. The actual grams of polymer in the conditioner differ from the total conditioner mass since other additives are added to the product to improve desired characteristics. This is certainly the case when using polymer emulsions that contain significant amounts of oil and water. Consequently, the grams of active polymer are often not reported, and the dose is calculated using the total weight of the product, not the active content. With the aim of knowing the actual polymer concentration, several methods have been developed to extract the active polymer from the additives in the conditioner (Herr and Routson 1974; Ghosh et al. 2010). For instance, STOWA (2017) developed a method to precipitate the active polymer by dissolving the conditioner in acetone. Acetone can dissolve salts, and the oil used in the commercial products, leaving only the active polymer.

A useful method to assess the suspension destabilisation is the use of the isoelectric point, which corresponds to the point at which the charge of the suspension is balanced, which is considered the minimum stability level (Agrawal et al. 1989). ZP and streaming current have been regarded as adequate tools to describe suspension destabilisation, since they describe the surface charge of particles when immersed in a liquid, and can be measured through simple tests (Dentel and Abu-Orf 1993; Byun et al. 2007). During ZP and streaming current assessment, proximity to zero is desired, since it resembles suspension instability; the instability window can be wider in the case of use of polymeric conditioners (Weiner et al. 1993; Clogston and Patri 2011; Marsalek 2014; Lunardi et al. 2021). Consequently, reaching the isoelectric point is not perse needed to achieve suspension destabilisation, and other mechanisms can destabilise the suspension without reaching the isoelectric point (López-Maldonado et al. 2014; Cano-Sarmiento et al. 2018). Furthermore, it is important to stress that other parameters such as pH or conductivity must also be considered to mimic full-scale conditions since they can also interfere with the suspension stability (Liao et al. 2002; Loell and Nanda 2018).

### Flocs formation

The phenomenon of floc formation is generally not evident in full-scale installations, particularly during centrifugation, where solid–liquid separation occurs almost immediately after mixing and destabilisation. However, in laboratory settings, floc formation can be observed during mixing. To optimise the dewatering process, stirring during mixing should promote floc growth rather than fragmentation, ensuring that flocs increase in size without breaking apart due to shear forces. Nonetheless, while this approach is suitable for controlled laboratory experiments, it does not accurately reflect the conditions in full-scale applications, where equipment such as centrifuges exerts significantly higher shear forces. This discrepancy is often overlooked by researchers, potentially limiting the relevance of lab-scale studies. Therefore, greater attention should be paid to replicating the mixing and shear conditions of full-scale equipment, such as centrifuges at the lab scale. This is particularly relevant when assessing the suitability of polymers, which must consider their shear resistance. For example, while optimal flocculation in the lab might suggest avoiding branched polymer types, these are commonly used in practice within centrifugation, highlighting the need for more representative testing conditions.

### Compression and expression

Different indexes differently address the phenomena of compression and expression. Nonetheless, the authors consider that these are the main overlooked phenomena during lab-scale dewaterability assessment. This is particularly relevant since these phenomena define the final solid´s content of the dewatered suspension, and the dismissal of any of them may result in inaccurate predictions.

**CST:** Easy to implement, simple to operate and cost-effective (if the standard devices are acquired). However, CST hardly allows to assess compression and expression phenomena and may not resemble the complexities of dewatering phenomena identified in Fig. 1 and thus may not provide accurate predictions (Coackley and Jones 1956; Meeten and Smeulders 1995). Compression and expression are not assessed since the capillary pressure exerted by the filtration medium is not comparable to the forces in full-scale processes. Also, it only allows to test a limited range of sludge concentrations, which may not necessarily be the concentration used in the full-scale process. Vesilind (1988) showed linear correlation with solids in concentration between 0.2–2% in the case of digested sludge and from 50–400 g/L in the case of an inert sludge, which does not necessarily correspond to the solids concentration expected in dewatering processes. Also, the hardly measurable short times measured when using conditioners challenge the establishment of significant differences between various conditioners or doses of these.

*Sedimentation methods:* Easy to implement, simple to operate and cost-effective. Techniques such as SVI and ZSV are more suitable for low solids concentration since sludges with high solids content may barely settle without the aid of an external force. These methods are perfectly suitable to assess the phenomena of suspension destabilisation and flocs formation. However, they cannot comparatively measure compression and expression, which hinders their use to assess dewatering processes.

*Centrifugation methods:* Once the centrifugation device is acquired, they are moderately easy to implement, with the accuracy of solids measurement being the main source of variability in the results. When applied properly, centrifugation methods result in an appropriate technique to assess the four phenomena described previously, including compression and expression. It is important to resemble the full-scale parameters in terms of equivalent force exerted (in RCF), and centrifugation time. The product of the RCF and the centrifugation time (RCF·t) can be used as a parameter to estimate the centrifugation intensity.

However, laboratory centrifugation does not fully reproduce the hydrodynamic conditions encountered in full-scale decanter centrifuges. Particularly, the configuration in lab-scale centrifugation does not reproduce shear and tumbling effects generated by the scroll conveyor in decanter centrifuges, which continuously transports and reworks the sludge cake. Furthermore, the sedimentation paths and solids transport mechanisms differ fundamentally between static batch centrifugation and the continuous flow regime in decanter centrifuges. As a result, the simple RCF·t parameter cannot fully represent the complex shear and hydrodynamic conditions experienced by sludge during full-scale centrifugation. Another notable drawback of conventional centrifugation methods is that the resulting cake remains submerged in water, meaning the pellet is always saturated. This can lead to an underestimation of the achievable dry matter content, which is a limitation for accurately simulating real dewatering processes. In contrast, the modified Higgins test allows water to escape the pellet, offering a more representative estimate of final cake dryness. Similarly, in full-scale operations such as belt filter presses or decanter centrifuges, additional mechanisms promote water removal which are not always saturated. For instance, in a decanter centrifuge, the sludge cake is conveyed across a “beach”, where it is compressed against the centrifugal force, separating more water. These differences should be considered when interpreting results from laboratory centrifugation. Additionally, in methods combining centrifugation and filtration, the pore size of the filtering cloth becomes critical to simulate full-scale conditions, just as in standard filtration-based tests.

*SRF:* Difficult to implement and operate. SRF measurements demand a filtration device with known a TMP, and a device to measure the permeate flow (commonly a scale), solids measurements, and a viscometer. In addition, trained human resources are needed to successfully conduct the assays. These conditions limit SRF use to research purposes or to installations with great analytical skills in which the protocols can be implemented and reproduced. Regarding the measurement, for incompressible cake samples, SRF allows quantitative comparisons when used under the same conditions. In the case of sewage sludge, cake compressibility hinders SRF use (Abboud and Corapcioglu 1993; Dominiak et al. 2011). This is particularly relevant since SRF is commonly measured using vacuum, and thus using a TMP that is around one order of magnitude lower than the TMP used to dewater suspensions in full-scale (Coackley and Jones 1956; Notebaert et al. 1975). In addition, the use of conditioners and time dependency of the technique may compromise the replicability of SRF as a technique to be used in lab-scale due to results disparity, which complicate to stablish significant differences among the tests. Therefore, it may not be suitable to assess dewaterability in compressible sludges or for comparison with full-scale results due to the overlooking of compression and expression.

*Other dead-end filtration methods:* Once the dead-end filtration device is acquired, the methods are moderately easy to implement and operate. As well as in the centrifugation methods, the main sources of operational difficulty arise from the solid’s measurements. When applied properly, dead-end filtration is an appropriate technique to assess the four phenomena described previously. However, it is important to resemble the full-scale parameters in terms of TMP, filtration time, and cloth pore size. Also, it is relevant to consider that the effluent quality may not have the same quality as the one in full-scale since part of the filtration is conducted using the cake as filtration medium, which may change over time. In addition, the cake compressibility may lead to discrepancies in the final solids content depending on the cake thickness, which may have to be adjusted to resemble full-scale results.

## Need for accurate dewatering assessment in the context of novel chemical conditioners

The arise of modern coagulants and flocculants forces the adoption of methodologies that properly assess their performance. The widespread use of organic flocculants at the industrial level has been subject of controversy in recent years. The controversy comes from the use of fossil fuels in their manufacturing processes, and the toxicity produced by their monomers or degradation products (Letterman and Pero 1990; Chang et al. 2005; Bolto and Gregory 2007; Nyyssölä and Ahlgren 2019). At present, the most frequently used flocculants are derived from polyacrylamide, produced through reactions involving reagents like acrylonitrile, propylene diallyl dimethyl ammonium chloride, vinyl benzyl trimethyl ammonium chloride, sulfonic acids, among others, depending on the desired outcome (Smith and Oehme 1991; Lentz 2015). Since these reagents do not originate from renewable sources, the production of polyacrylamide contributes to atmospheric CO_2_ emissions (Arcadis Nederland B.V. 2024).

In this context, two primary approaches have been identified to enhance the environmental sustainability of organic flocculants: firstly, utilising non-fossil feedstocks throughout the manufacturing chain of existing flocculants; and secondly, developing a new set of flocculants based on renewable biopolymers. The first approach grounds on the flocculant's commercial presentations i.e., powder, water-based dispersion, and oil-based emulsions. In terms of CO_2_ emissions, flocculants based on inverse emulsion polymerisation (oil emulsions) are particularly significant, as their polymer content represents only 20–60% of the total emulsion (Graillat et al. 1986; Cheng et al. 2019; Yang et al. 2020), with the remainder being a carbon-based solvent. The second approach to overcome the current use of organic flocculants are the “so called” natural flocculants or bio-based flocculants. These polymers comprise compounds such as starch, cellulose, guar gum, alginates, pullulan, pectin, chitosan, and gelatine (Singh et al. 2003; Garcia et al. 2009; Salehizadeh et al. 2018; Blockx et al. 2021) may replace the flocculants currently in use.

Lack of predictability observed when assessing dewaterability for known matrices and conditioners may be augmented in the case of poorly known conditioners. Currently the methods used to assess performance of new conditioners describe physicochemical characteristics of these compounds or the formerly mentioned indexes (Hu et al. 2021; Tian et al. 2025). However, the aim is to substitute the chemicals that are currently used in full-scale, which, to the best knowledge of the authors has not been massively reached. Therefore, the early proposed considerations may help to assess the effectiveness of novel, more sustainable conditioners that do not behave in a similar way to traditional polyacrylamide-based flocculants.

## Conclusions

This review allows us to draw the following conclusions:

Despite the existence of known characteristics of sludge and conditioners that allow to predict dewaterability performance, matrix variability challenges the comparison of lab and full-scale methods. Consequently, practical indexes like SRF, CST, centrifugation, or sedimentation are used to predict full-scale dewaterability. These indexes do not consider all the complexities of the process since not all the proposed phenomena of the dewatering process are commonly assessed in the techniques used to assess dewaterability in lab-scale.

A phenomenological analysis of sludge dewatering indicates that four processes are fundamental for dewaterability assessment: (1) mixing of sludge and conditioner, (2) suspension destabilisation, (3) floc formation, and (4) compression and expression. The comparative assessment of commonly applied indexes provide limited insights into compression and expression, which ultimately determine the achievable solids concentration. Methods based on combined centrifugation and filtration showed greater potential to capture these later stages, increasing predictability.

The rise of novel, more sustainable conditioners challenges the existing methods to assess dewaterability and improve predictability. Adequate methods to analyse dewaterability must consider the four proposed phenomena of the dewatering process to overcome the biases of traditional methods.

Despite the extensive information in the field, the literature analysis identified the following research gaps: (1) Need for improved experimental methodologies to better characterise sludge compressibility and its impact on water release during the compression and expression phases, (2) development of standardised testing protocols capable of linking laboratory measurements with full-scale dewatering performance; (3) increasing interest in environmentally friendly and bio-based conditioners requires assessment methods capable of evaluating their performance under variable sludge matrix conditions.

## Data Availability

No datasets were generated or analysed during the current study.

## Abbreviations

CST: Capillary suction time DI drainage index
DI: Drainage index
DLVO: Deryagin–Landau–Verwey–Overbeek
G: Mean velocity gradient
SRF: Specific resistance to filtration
SVI: Sludge volumetric index
t: Time
TMP: Trans membrane pressure
ZP: Zeta potential
ZSV: Zone settling velocity
